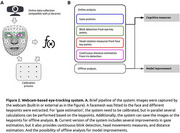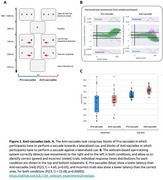# Remote Eye‐Tracking for Cognitive Science and Health Applications: Validating the Anti‐Saccade Task in a Web‐Based Setting

**DOI:** 10.1002/alz70856_107106

**Published:** 2026-01-13

**Authors:** Gustavo E Juantorena, Agustín Penas, Francisco Figari, Agustin Petroni, Juan E Kamienkowski

**Affiliations:** ^1^ Facultad de Ciencias Exactas y Naturales, Universidad de Buenos Aires, Ciudad Autonoma de Buenos Aires, CABA, Argentina; ^2^ Instituo de Ciencias de la Computacion (CONICET‐UBA), Ciudad Autonoma de Buenos Aires, Ciudad Autonoma de Buenos Aires, Argentina; ^3^ Facultad de Ciencias Exactas y Naturales, Universidad de Buenos Aires, Ciudad Autonoma de Buenos Aires, Ciudad Autonoma de Buenos Aires, Argentina; ^4^ University of Gothenburg, Gothenburg, Gothenburg, Sweden

## Abstract

**Background:**

In recent years, several prototypes of remote, webcam‐based eye‐tracking have emerged, exploring their feasibility and potential for web‐based experiments. This growing interest is largely driven by the ability to conduct tasks remotely, enabling research on larger and hard‐to‐reach populations. However, its use has been primarily limited to proof‐of‐concept studies in basic cognitive science. These studies have generally reported a decrease in precision, compounded by lower camera quality and noisier environments, posing new implementation challenges. Additionally, webcam‐based eye‐tracking has been applied in human‐computer interaction and marketing, where only qualitative results are typically required. Here, we aim to extend its application to cognitive tasks relevant to mental health and telemedicine.

**Methods:**

We present a novel prototype of a remote, webcam‐based eye‐tracker with key enhancements over existing systems, including a reliable sampling rate, screen distance detection, head movement tracking, blink detection, and improved calibration without requiring constant mouse interactions. We first evaluated its spatiotemporal resolution and reliability. Next, we tested its functionality in a cognitive experiment using the anti‐saccade task, a well‐established paradigm in various patient populations. This task assesses inhibitory control by comparing horizontal eye movements toward (pro‐saccades) and away from (anti‐saccades) a target.

**Results:**

The proposed improvements resulted in a robust remote eye‐tracking system that performed consistently across different hardware setups and maintained stable calibration over time. We also introduced novel capabilities and provided a discussion on their limitations. Furthermore, our results replicated key findings from high‐precision laboratory‐based eye‐trackers: response times and error rates were higher in anti‐saccades than in pro‐saccades, and incorrect responses were associated with shorter reaction times.

**Conclusions:**

We demonstrate the potential of this prototype for both cognitive research and clinical applications, providing a comprehensive evaluation of its capabilities and limitations. Our findings indicate that remote webcam‐based eye‐tracking can successfully replicate classical results from the anti‐saccade task in an online setting.